# Supplementary material for: Trend and Joinpoint Analysis of Cancer Incidence and 1-Year Mortality in North-East Spain 2005–2020
Source: Cancers (Basel). 2023 Nov 22;15(23):5527. doi: 10.3390/cancers15235527 (PMC10705763; doi:10.3390/cancers15235527)
Supplement: Supplementary file 1 [file cancers-15-05527-s001.zip › cancers-2663994-supplementary.pdf]

SUPPLEMENTARY MATERIALS

Supplementary Figures: Pages 2-5

Supplementary Methods: Page 6

Supplementary Tables: Pages 7-14

SUPPLEMENTARY FIGURES

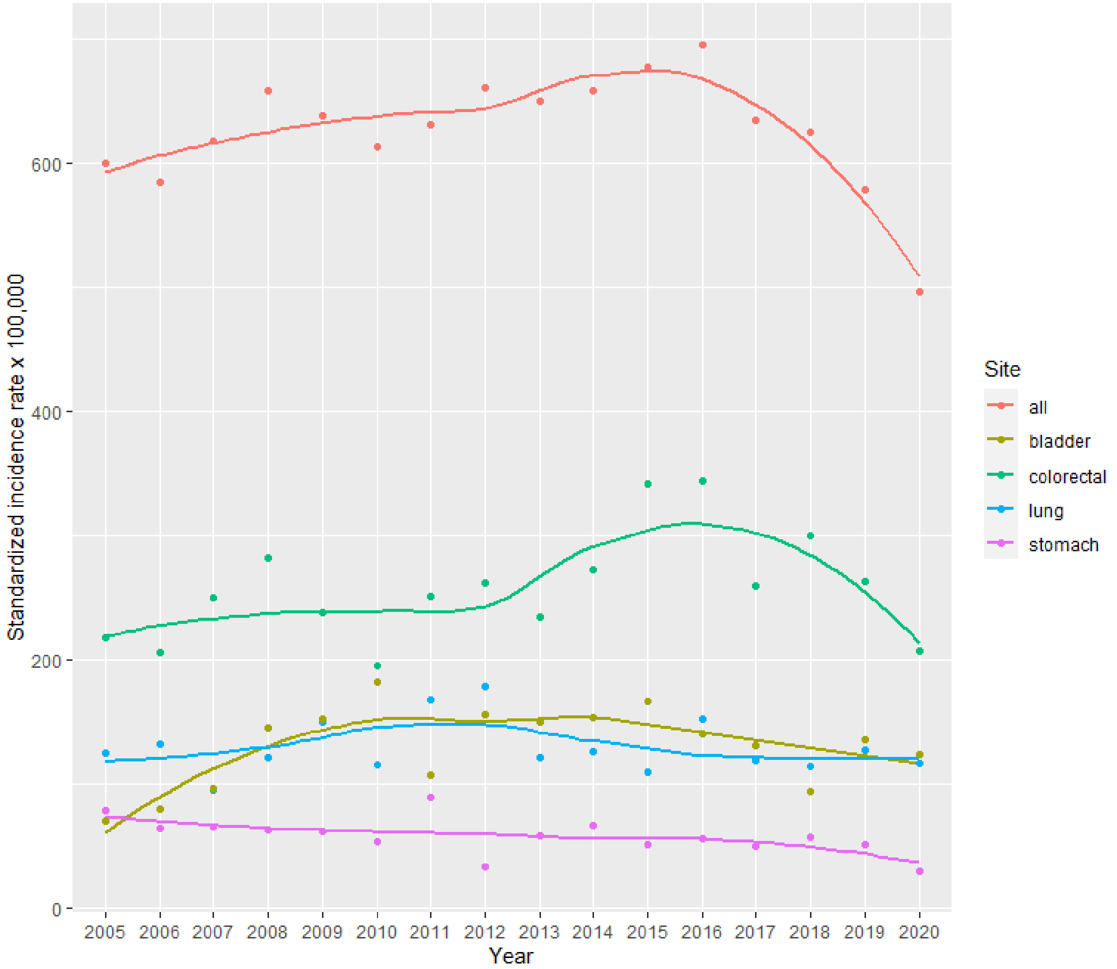

Figure S1. Cancer age-standardized incidence by cancer localization in females and males together from Osona during 2005-2020. Values are presented as dots. Trends are shown with a smooth local regression line. For age-standardization we used the 2013 European Standard Population.

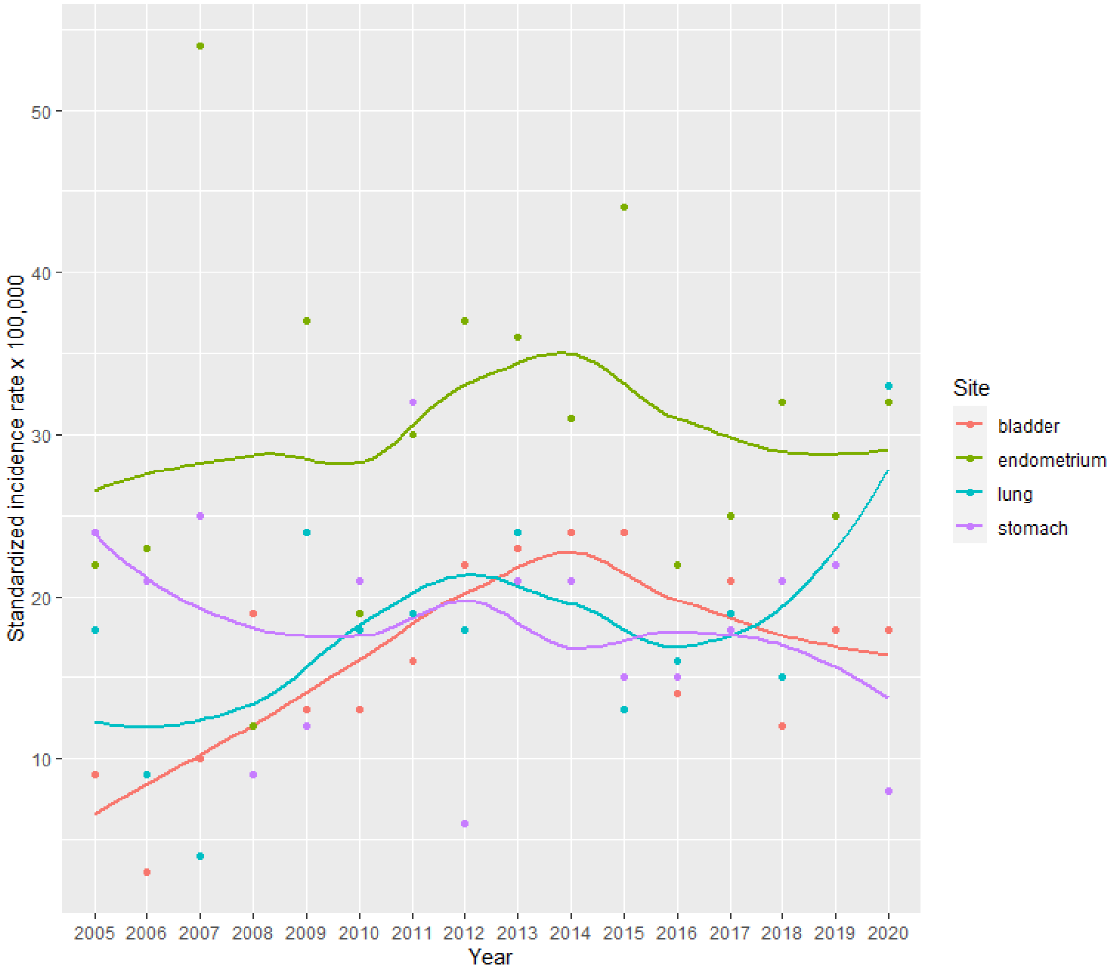

Figure S2. Cancer age-standardized incidence by cancer localization in females from Osona during 2005-2020. Values are presented as dots. Trends are shown with a smooth local regression line. For age-standardization we used the 2013 European Standard Population.

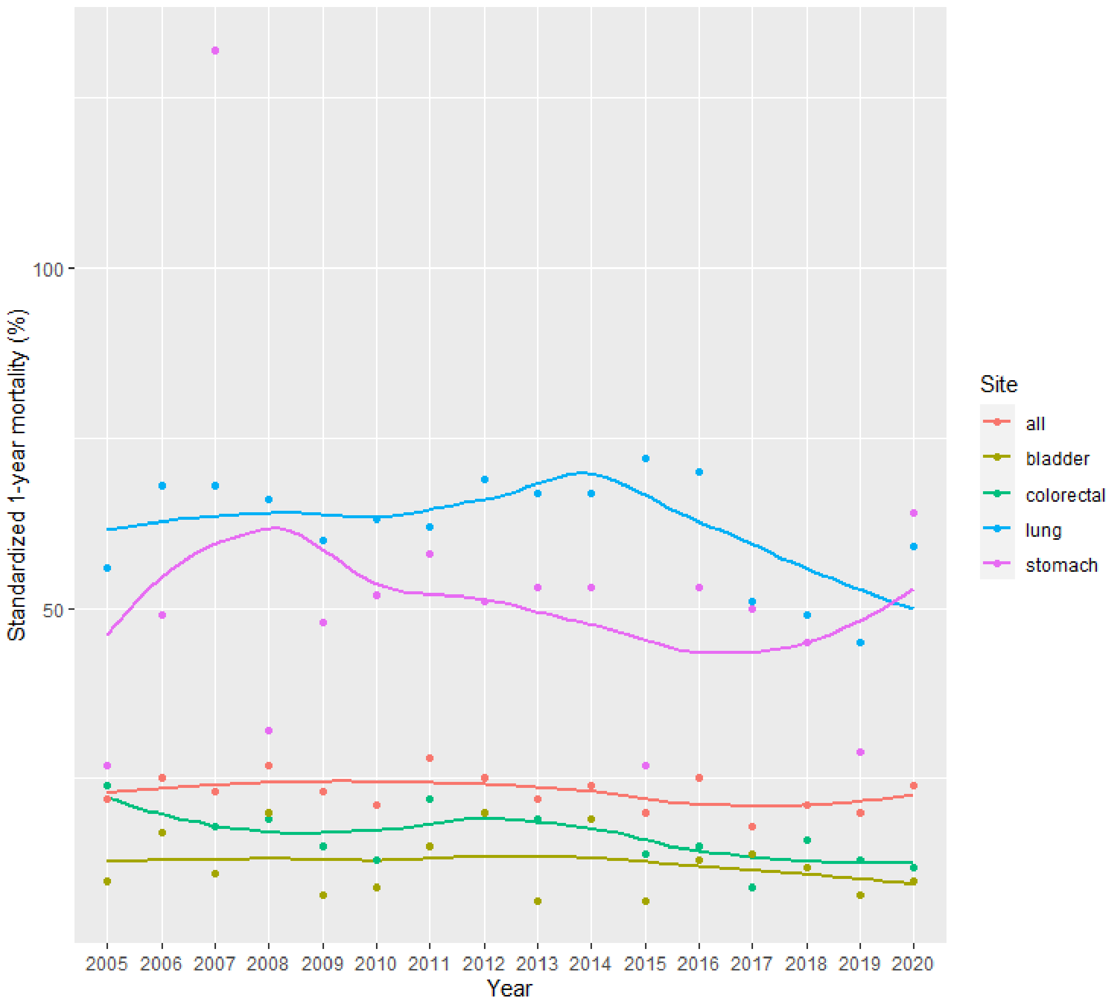

Figure S3. Cancer age-standardized 1-year mortality by cancer localization in females and males together from Osona during 2005-2020. Values are presented as dots. Trends are shown with a smooth local regression line. For age-standardization we used the survival weights from the Surveillance, Epidemiology, and End Results Program of the US National Cancer Institute.

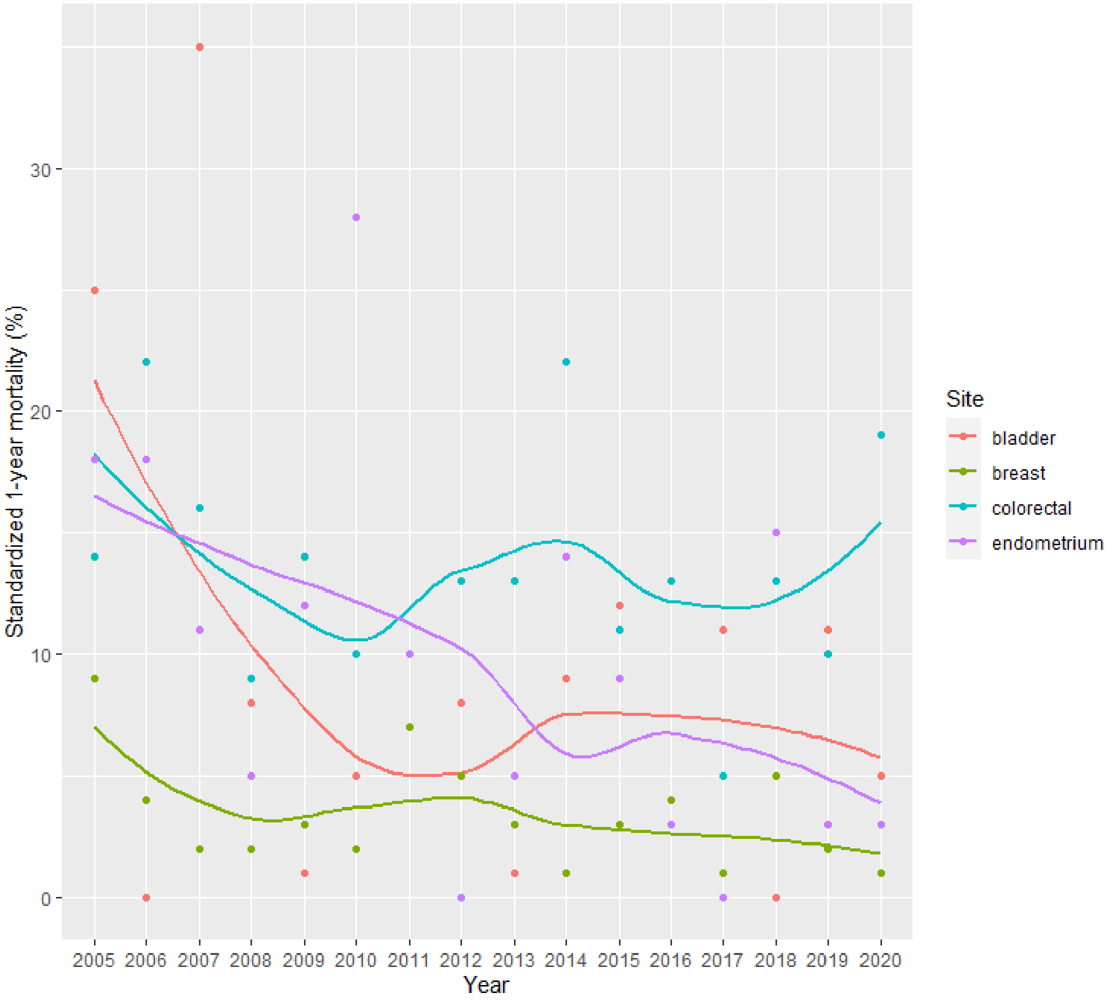

Figure S4. Cancer age-standardized 1-year mortality by cancer localization in females from Osona during 2005-2020. Values are presented as dots. Trends are shown with a smooth local regression line. For age-standardization we used the survival weights from the Surveillance, Epidemiology, and End Results Program of the US National Cancer Institute.

SUPPLEMENTARY METHODS

Permutation test

The permutation test is used for testing between two models with increasing number of joinpoints. To test this a ratio is calculated between the sum of squared errors from the null model and sum of squared errors from the alternative model with joinpoints. If the ratio is close to 1, models are similar, if the ratio is large the alternative model is better. The permutation method is used to define how large the ratio has to be for identification as statistically significant. In this method, the residuals from the null model are permuted, and permutation data sets are created. Then, the described ratios are calculated in the permuted datasets. And the following proportion (p-value) is calculated: proportion of permutation datasets in which the ratio values are as extreme as those in the original dataset. If the model with no joinpoints is correct, half of the ratios from the permutation datasets would be larger than the ratio from the original data. On the other hand, if the model with a joinpoint is correct, most of the ratios from the permutation datasets would be lower than the ratio from the original data and the p-value would be small.

SUPPLEMENTARY TABLES

Table S1. Crude incidence rates (/100.000 inhabitants) of the most common cancer sites in females and males from Osona in 2005-2020.

Year	All sites	Colorectal	Lung	Bladder	Stomach
2005	527	169	96	54	60
2006	520	157	97	62	50
2007	547	197	75	78	50
2008	581	217	95	113	50
2009	565	187	118	114	45
2010	545	156	92	135	39
2011	574	210	138	84	68
2012	605	212	142	122	29
2013	600	196	101	125	47
2014	613	229	106	129	57
2015	635	297	91	135	45
2016	665	303	133	123	51
2017	606	228	109	109	46
2018	596	261	98	82	49
2019	560	231	111	117	46
2020	482	180	100	107	27

Table S2. Crude incidence rates (/100.000 inhabitants) of the most common cancer sites in females from Osona in 2005-2020.

Year	All sites	Colorectal	Lung	Bladder	Stomach	Breast	Endometrium
2005	414	49	17	8	24	171	20
2006	426	48	10	3	21	180	21
2007	457	83	5	13	21	166	48
2008	429	78	9	21	9	136	11
2009	464	77	22	12	14	147	35
2010	450	56	17	14	20	189	17
2011	472	80	19	16	32	158	28
2012	503	73	17	22	6	166	35
2013	521	81	22	27	20	162	33
2014	561	95	22	25	23	188	30
2015	530	109	12	23	17	175	42
2016	566	126	17	17	20	202	23
2017	517	75	18	22	18	199	25
2018	519	101	14	10	21	168	32
2019	471	93	19	19	24	153	26
2020	478	66	33	21	9	136	31

Table S3. Crude incidence rates (/100.000 inhabitants) of the most common cancer sites in males from Osona in 2005-2020.

Year	All sites	Colorectal	Lung	Bladder	Stomach	Prostate
2005	639	120	78	46	36	161
2006	614	109	87	58	28	149
2007	637	113	70	65	28	177
2008	733	138	85	92	41	133
2009	666	109	96	101	30	149
2010	641	99	74	120	17	125
2011	677	128	119	68	35	148
2012	708	137	124	99	22	129
2013	681	112	78	98	26	150
2014	668	130	83	103	32	135
2015	744	184	78	111	27	137
2016	766	173	115	105	30	134
2017	699	151	90	86	27	140
2018	674	158	83	71	26	166
2019	649	136	91	97	21	139
2020	547	113	66	86	18	107

Table S4. Age-standardized incidence rates (/100.000 inhabitants) of the most common cancer sites in females and males from Osona in 2005-2020.

Year	All sites	Colorectal	Lung	Bladder	Stomach
2005	600	218	125	70	79
2006	585	206	132	80	64
2007	618	250	95	97	66
2008	659	283	122	146	63
2009	639	238	150	153	62
2010	614	196	116	183	54
2011	632	252	168	108	89
2012	661	262	179	156	33
2013	651	235	122	150	59
2014	659	273	126	154	67
2015	678	342	110	167	51
2016	696	345	153	141	56
2017	635	260	119	131	50
2018	626	300	114	94	57
2019	579	263	128	136	51
2020	497	207	117	124	30

The 2013 European Standard Population was used for age-standardization.

Table S5. Age-standardized incidence rates (/100.000 inhabitants) of the most common cancer sites in females from Osona in 2005-2020.

Year	All sites	Colorectal	Lung	Bladder	Stomach	Breast	Endometrium
2005	440	51	18	9	24	189	22
2006	437	49	9	3	21	187	23
2007	477	88	4	10	25	174	54
2008	444	79	9	19	9	148	12
2009	479	76	24	13	12	162	37
2010	465	54	18	13	21	201	19
2011	486	80	19	16	32	165	30
2012	512	73	18	22	6	167	37
2013	517	79	24	23	21	162	36
2014	557	91	24	24	21	191	31
2015	533	111	13	24	15	177	44
2016	548	125	16	14	15	199	22
2017	502	71	19	21	18	197	25
2018	506	101	15	12	21	163	32
2019	448	90	18	18	22	150	25
2020	405	63	33	18	8	131	32

The 2013 European Standard Population was used for age-standardization.

Table S6. Age-standardized incidence rates (/100.000 inhabitants) of the most common cancer sites in males from Osona in 2005-2020.

Year	All sites	Colorectal	Lung	Bladder	Stomach	Prostate
2005	836	157	104	59	46	218
2006	784	142	115	75	36	193
2007	810	147	88	80	34	231
2008	940	178	109	120	51	180
2009	852	134	121	131	46	199
2010	813	122	95	160	25	164
2011	840	156	145	86	47	193
2012	870	169	156	125	26	167
2013	836	135	94	118	32	198
2014	802	155	100	124	38	170
2015	868	208	94	135	30	168
2016	883	193	135	122	34	163
2017	811	176	99	104	29	170
2018	776	183	98	80	31	196
2019	745	154	104	114	25	164
2020	621	130	79	100	20	125

The 2013 European Standard Population was used for age-standardization.

Table S7. Age-standardized 1-year mortality (%) due to the most common cancer sites in females and males from Osona in 2005-2020.

Year	All sites	Colorectal	Lung	Bladder	Stomach
2005	22	24	56	10	27
2006	25	17	68	17	49
2007	23	18	68	11	132
2008	27	19	66	20	32
2009	23	15	60	8	48
2010	21	13	63	9	52
2011	28	22	62	15	58
2012	25	20	69	20	51
2013	22	19	67	7	53
2014	24	19	67	19	53
2015	20	14	72	7	27
2016	25	15	70	13	53
2017	18	9	51	14	50
2018	21	16	49	12	45
2019	20	13	45	8	29
2020	24	12	59	10	64

For age-standardization, we used the survival weights from the Surveillance, Epidemiology, and End Results Program of the US National Cancer Institute.

Table S8. Age-standardized 1-year mortality (%) due to the most common cancer sites in females from Osona in 2005-2020.

Year	All sites	Colorectal	Lung	Bladder	Stomach	Breast	Endometrium
2005	19	14	38	25	29	9	18
2006	20	22	43	0	15	4	18
2007	19	16	25	35	94	2	11
2008	20	9	38	8	25	2	5
2009	20	14	35	1	33	3	12
2010	19	10	48	5	45	2	28
2011	23	10	51	7	61	7	10
2012	17	12	81	8	28	5	0
2013	18	13	55	1	44	3	5
2014	20	22	58	9	49	1	14
2015	17	11	74	12	16	3	9
2016	18	13	19	4	28	4	3
2017	13	5	56	11	23	1	0
2018	19	13	44	0	37	5	15
2019	17	10	44	11	19	2	3
2020	19	19	42	5	47	1	3

For age-standardization, we used the survival weights from the Surveillance, Epidemiology, and End Results Program of the US National Cancer Institute.

Table S9. Age-standardized 1-year mortality (%) due to the most common cancer sites in males from Osona in 2005-2020.

Year	All sites	Colorectal	Lung	Bladder	Stomach	Prostate
2005	25	25	59	8	26	6
2006	29	17	65	18	75	11
2007	26	14	69	8	31	1
2008	32	24	66	22	36	3
2009	26	13	65	7	48	2
2010	23	12	66	8	32	3
2011	32	28	65	17	40	7
2012	31	24	64	20	49	2
2013	26	24	69	8	37	6
2014	27	18	70	21	43	5
2015	24	17	70	5	35	10
2016	29	14	65	14	50	6
2017	23	11	50	15	75	8
2018	23	18	48	13	38	2
2019	23	12	45	5	21	3
2020	29	8	66	10	72	7

For age-standardization, we used the survival weights from the Surveillance, Epidemiology, and End Results Program of the US National Cancer Institute.

Table S10. Sensitivity analyses of standardized incidence rates joinpoint regression with log-linear models assuming autocorrelated errors.

	Correlation 0.1		Correlation 0.2		Correlation 0.3	
	Period	APC (95% CI)	Period	APC (95% CI)	Period	APC (95% CI)
**Both sexes**						
All Cancers	2005-2016	1.3(0.5,2.1)	2005-2016	1.3(0.5,2.2)	2005-2016	1.3(0.4,2.3)
	2016-2020	-6.7(-9.6,-3.6)	2016-2020	-6.9(-10.0,-3.6)	2016-2020	-7.0(-10.3,-3.5)
Colorectal	2005-2016	3.8(0.7,7.7)	2005-2016	3.9(0.5,7.3)	2005-2016	3.9(0.3,7.7)
	2016-2020	-8.1(-19.4,4.7)	2016-2020	-8.5(-20.0,4.7)	2016-2020	-8.9(-20.6,4.6)
Lung and bronchus	2005-2020	-0.4(-2.6,1.9)	2005-2020	-0.4(-2.9,2.1)	2005-2020	-0.5(-3.2,2.4)
Stomach	2005-2020	-3.0(-5.7,-0.3)	2005-2020	-3.1(-6.2,0.1)	2005-2020	-3.2(-6.8,0.5)
Urinary bladder	2005-2009	25.2(3.3,51.7)	2005-2009	25.1(2.2,53.1)	2005-2009	19.0(2.8,37.8)
	2009-2020	-2.6(-6.0,0.8)	2009-2020	-2.6(-6.3,1.2)	2009-2020	-3.5(-8.1,1.2)
**Females**						
All Cancers	2005-2016	2.3(1.4,3.1)	2005-2016	2.3(1.3,3.2)	2005-2016	2.2(1.1,3.4)
	2016-2020	-7.0(-10.4,-3.5)	2016-2020	-7.0(-10.6,-3.3)	2016-2020	-7.1(-10.9,-3.1)
Breast	2005-2020	-0.8(-2.2,0.7)	2005-2020	-0.8(-2.4,0.8)	2005-2020	-0.9(-2.7,0.8)
Colorectal	2005-2016	6.0(1.1,11.0)	2005-2016	6.1(0.8,11.7)	2005-2016	6.3(0.4,12.5)
	2016-2020	-9.9(-26.5,10.6)	2016-2020	-10.4(-27.5,10.8)	2016-2020	-11.0(-28.7,11.0)
Endometrium	2005-2020	-0.6(-5.0,3.9)	2005-2020	-0.9(-5.9,4.3)	2005-2020	-1.3(-6.9,4.6)
Lung and bronchus	2005-2020	3.6(-0.8,8.1)	2005-2020	3.6(-1.1,8.4)	2005-2020	3.5(-1.5,8.8)
Stomach	2005-2020	-1.7(-6.2,3.1)	2005-2020	-1.8(-6.9,3.5)	2005-2020	-2.0(-7.8,4.1)
Urinary bladder	2005-2020	3.2(-1.2,7.8)	2005-2020	3.1(-1.6,8.0)	2005-2020	3.0(-2.1,8.3)
**Males**						
All Cancers	2005-2016	0.4(-0.8,1.7)	2005-2016	0.4(-0.9,1.8)	2005-2016	0.4(-1.0,2.0)
	2016-2020	-6.7(-11.7,-1.5)	2016-2020	-6.9(-12.0,-1.4)	2016-2020	-7.0(-12.4,-1.3)
Colorectal	2005-2020	0.8(-1.1,2.8)	2005-2020	0.7(-1.4,2.9)	2005-2020	0.6(-1.7,3.0)
Lung and bronchus	2005-2020	-1.0(-3.5,1.6)	2005-2020	-1.0(-3.8,1.9)	2005-2020	-1.0(-4.2,2.2)
Prostate	2005-2020	-2.1(-3.4,-0.7)	2005-2020	-2.1(-3.6,-0.6)	2005-2020	-2.2(-4.0,-0.4)
Stomach	2005-2020	-3.7(-6.1,-1.2)	2005-2020	-3.8(-6.6,-0.9)	2005-2020	-3.9(-7.2,-0.6)
Urinary bladder	2005-2009	23.7(0.9,51.6)	2005-2009	23.7(0.9,51.6)	2005-2009	23.7(0.9,51.6)
	2009-2020	-2.8(-6.4,0.8)	2009-2020	-2.8(-6.4,0.8)	2009-2020	-2.8(-6.4,0.8)

Analyses were done with the Joinpoint Regression Program. APC: annual percentage change; CI: confidence interval.

Table S11. Sensitivity analyses of standardized 1-year mortality joinpoint regression with log-linear models assuming autocorrelated errors.

	**Correlation 0.1**		**Correlation 0.2**		**Correlation 0.3**	
	Period	APC (95% CI)	Period	APC (95% CI)	Period	APC (95% CI)
**Both sexes**						
All Cancers	2005-2020	-0.9(-2.3,0.5)	2005-2020	-0.8(-2.4,0.7)	2005-2020	-0.8(-2.6,1.1)
Colorectal	2005-2020	-3.1(-5.3,-0.8)	2005-2020	-3.1(-5.6,-0.6)	2005-2020	-3.1(-5.9,-0.3)
Lung and bronchus	2005-2020	-1.0(-2.7,0.6)	2005-2020	-1.0(-2.7,0.8)	2005-2020	-0.8(-2.7,1.1)
Stomach	2005-2020	0.1(-5.1,15.7)	2005-2020	0.5(-5.4,6.8)	2005-2020	0.9(-5.8,8.2)
Urinary bladder	2005-2020	-0.8(-5.4,3.9)	2005-2020	-0.8(-5.9,4.7)	2005-2020	-0.6(-6.6,5.8)
**Females**						
All Cancers	2005-2020	-1.1(-2.5,0.4)	2005-2020	-1.0(-2.7,0.7)	2005-2020	-0.9(-2.8,1.1)
Breast	2005-2020	-4.2(-10.0,1.9)	2005-2020	-4.3(-10.6,2.4)	2005-2020	-4.3(-11.1,3.0)
Colorectal	2005-2020	-0.1(-4.3,4.3)	2005-2020	0.2(-4.5,5.2)	2005-2020	0.5(-4.8,6.2)
Endometrium	2005-2020	---	2005-2020	---	2005-2020	---
Lung and bronchus	2005-2020	0.3(-4.9,5.7)	2005-2020	0.3(-5.5,6.3)	2005-2020	0.2(-6.2,7.1)
Stomach	2005-2020	-2.8(-9.5,4.4)	2005-2020	-2.9(-10.5,5.2)	2005-2020	-3.2(-11.7,6.1)
Urinary bladder	2005-2020	---	2005-2020	---	2005-2020	---
**Males**						
All Cancers	2005-2020	-0.7(-2.2,0.8)	2005-2020	-0.6(-2.3,1.1)	2005-2020	-0.6(-2.5,1.4)
Colorectal	2005-2020	-3.4(-6.9,0.3)	2005-2020	-3.5(-7.3,0.5)	2005-2020	-3.6(-7.9,0.8)
Lung and bronchus	2005-2015	1.3(0.2,2.5)	2005-2020	1.4(0.2,2.6)	2005-2020	-1.0(-2.8,0.8)
	2015-2018	-13.7(-23.6,-2.5)	2015-2018	-13.7(-23.0,-3.1)		
	2018-2020	15.2(-0.9,33.8)	2018-2020	15.2(0.0,32.8)		
Prostate	2005-2020	2.1(-4.2,8.8)	2005-2020	2.2(-4.6,9.5)	2005-2020	2.4(-5.0,10.4)
Stomach	2005-2020	2.6(-1.8,7.2)	2005-2020	3.0(-2.1,8.3)	2005-2020	3.5(-2.3,9.6)
Urinary bladder	2005-2020	-0.7(-6.9,5.9)	2005-2020	-0.6(-7.7,7.0)	2005-2020	-0.5(-8.6,8.4)

Analyses were done with the Joinpoint Regression Program. Analyses could not be performed for endometrium and urinary bladder cancer in females due to the low number of events. APC: annual percentage change; CI: confidence interval.

Table S12. Sensitivity analyses of standardized incidence rates and 1-year mortality joinpoint regression with generalized linear models and Davies test.

	Period	APC(95% CI)	Period	APC(95% CI)
Incidence				
**Both sexes**				
All cancers	2005-2016	7.8(4.6,1.1)	2016-2020	**-4.6(-6.3,-2.9)**
Colorectal	2005-2016	**9.3(3.5,15.0)**	2016-2020	-20.5(-51.2,10.2)
Urinary bladder	2005-2009	**2.3(10.0,3.6)**	2009-2020	-3.8(-7.8,1.6)
**Females**				
All cancers	2005-2016	**11.0(7.9,14.1)**	2016-2020	**-34.0(-44.3,-23.7)**
Breast	2005-2017	1.1(-1.3,3.5)	2017-2020	-18.1(-40.7,4.5)
Colorectal	2005-2016	**4.6(1.5,7.6)**	2016-2020	-8.2(-18.4,2.0)
Urinary bladder	2005-2013	**2.0(9.7,3.0)**	2013-2020	-1.1(-2.6,3.5)
**Males**				
All cancers	2005-2016	2.7(-4.2,9.6)	2016-2020	**-60.0(-96.8,-23.4)**
Urinary bladder	2005-2009	**1.9(7.3,30.5)**	2009-2020	-3.2(-6.7,0.3)
1-year mortality				
**Both sexes**				
Lung and bronchus	2005-2015	0.6(-0.8,1.9)	2015-2020	**-4.0(-7.0,-1.0)**
**Females**				
Breast	2005-2016	-5.0(-10.1,0.1)	2015-2016	-0.1(-0.3,0.2)

Analyses were done with the R Statistical Software. APC: annual percentage change; CI: confidence interval.
